# “I don’t see the whole picture of their health”: a critical ethnography of constraints to interprofessional collaboration in end-of-life conversations in primary care

**DOI:** 10.1186/s12875-023-02171-w

**Published:** 2023-10-28

**Authors:** Celina Carter, Shan Mohammed, Ross Upshur, Pia Kontos

**Affiliations:** 1 Dalla Lana School of Public Health, University of Toronto, Toronto, Canada; 2 Lawrence S. Bloomberg Faculty of Nursing, University of Toronto, Toronto, Canada; 3 Department of Family and Community Medicine and Dalla Lana School of Public Health, University of Toronto, Toronto, Canada; 4 KITE Research Institute, Toronto Rehabilitation Institute - University Health Network, Toronto, Canada

**Keywords:** Frailty, Primary care, Interprofessional collaboration, End-of-life conversations, Goals-of-care, Nursing, Allied health clinicians, Biomedical dominance

## Abstract

**Context:**

Interprofessional collaboration is recommended in caring for frail older adults in primary care, yet little is known about how interprofessional teams approach end-of-life (EOL) conversations with these patients.

**Objective:**

To understand the factors shaping nurses’ and allied health clinicians’ involvement, or lack of involvement in EOL conversations in the primary care of frail older adults.

**Methods/setting:**

A critical ethnography of a large interprofessional urban Family Health Team in Ontario, Canada. Data production included observations of clinicians in their day-to-day activities excluding direct patient care; one-to-one semi-structured interviews with clinicians; and document review. Analysis involved coding data using an interprofessional collaboration framework as well as an analysis of the normative logics influencing practice.

**Participants:**

Interprofessional clinicians (*n* = 20) who cared for mildly to severely frail patients (Clinical Frailty Scale) at the Family Health Team.

**Results:**

Findings suggest primary care nurses and allied health clinicians have the knowledge, skills, and inclination to engage frail older adults in EOL conversations. However, the culture of the clinic prioritizes biomedical care, and normalizes nurses and allied health clinicians providing episodic task-based care, which limits the possibility for these clinicians’ engagement in EOL conversations. The barriers to nurses’ and allied health clinicians’ involvement in EOL conversations are rooted in neoliberal-biomedical ideologies that shapes the way primary care is governed and practiced.

**Conclusions:**

Our findings help to explain why taking an individual-level approach to addressing the challenge of delayed or avoided EOL conversations, is unlikely to result in practice change. Instead, primary care teams can work to critique and redevelop quality indicators and funding models in ways that promote meaningful interprofessional practice that recognize the expertise of nursing and allied health clinicians in providing high quality primary care to frail older patients, including EOL conversations.

**Supplementary Information:**

The online version contains supplementary material available at 10.1186/s12875-023-02171-w.

## Background

Interprofessional collaboration is central to the primary care of complex chronic illnesses such as medical frailty [1, 2]. To support medically frail older patients, who often have complex multimorbidity and an increased risk of mortality [3], the interprofessional team should collectively engage patients and their care partners in end-of-life (EOL) conversations [4–7]. Engaging in talk about immediate goals, fears, and wishes when facing a life-limiting illness can improve quality-of-life and goal-concordant care [8]. However, little research has explored the forces that shape interprofessional collaboration to support EOL conversations in primary care. To address this knowledge gap, which limits the ability to improve practice, we conducted a critical ethnography to examine the structural forces shaping nurses’ and allied health clinicians’ involvement in EOL conversations in the primary care of frail older adults.

### Interprofessional primary care

Primary care is the first point of contact with the healthcare system. It provides longitudinal care and aims to prevent and manage chronic illnesses, which is best achieved by an interprofessional approach that involves sharing knowledge, skills, and experience [9]. As primary care grapples with increasingly complex multi-morbid patients, who are discharged from acute care “quicker and sicker” and require comprehensive care from a team, interprofessional collaboration is essential [1, 10]. Interprofessional collaboration is defined as a partnership between a team of clinicians as well as a patient in “a participatory, collaborative and coordinated approach to shared decision-making around health and social issues” [11] (p.1). Effective collaboration requires understanding of team members’ roles, effectively managing conflict, supporting team functioning, and collaboratively formulating, implementing, and evaluating care to enhance patient outcomes [11]. In primary care, the most responsible provider, usually a physician or nurse practitioner, is considered the medical expert. Within interprofessional teams, the most responsible provider has an overall responsibility for directing and coordinating patients’ care [12–14]. At the same time, most responsible providers are expected to understand, respect, and support their overlapping roles and responsibilities with other clinicians on the team and be able to change from team leader to team member based on need [15].

Primary care exists within larger healthcare systems and social policies, which in Canada and other countries internationally tend to be shaped by neoliberal logics [16] that promote cost and speed efficiencies, government deregulation to encourage innovation, privatization of public services to lower costs for the state, and individual responsibility to offload structural problems onto individuals [17–20]. Research shows that healthcare systems governed by neoliberalism are often characterized by interprofessional isolation and conflict as opposed to effective collaboration [16, 21, 22]. To date, little work has explored the connection between social policies, interprofessional collaboration, and EOL conversations.

### End-of-life conversations in primary care

As patients approach their EOL, having a clear understanding of their wishes and values and aligning these to care becomes essential for person-centred EOL care [12]. This can be accomplished through EOL conversations, which include three types of conversations: 1) advance care planning; 2) goals-of-care discussions; and 3) EOL decision-making discussions [23, 24]. The focus in this study is on the latter two types of EOL conversations since research has found that advance care planning does not improve EOL care, influence EOL decision-making, help to align care with patient goals, or improve satisfaction of care [25, 26].

EOL goals-of-care and decision-making discussions require providing information about the illness as well as the harms and benefits of medical interventions, exploring what matters to patients and their care partners, and making recommendations based on expressed goals [23]. Determining goals-of-care is considered the “gold standard” for ensuring person-centred conversations and decision-making [27]. However, research has found that physicians often do not engage patients in EOL decision-making and goals-of-care discussions until death is imminent [4, 28]. To address this, an interprofessional approach is recommended for achieving timely and high-quality EOL conversations in primary care [4, 28]. If physicians and nurse practitioners collaborate interprofessionally, alignment between patients’ goals and the care provided in the last years, months, or weeks of life might improve [13].

### Nursing and allied health clinicians’ roles in EOL conversations

According to the Ontario Palliative Care Competency Framework (2019), it is well within the scope of nurses, social workers, occupational therapists, physiotherapists, pharmacists and many other clinicians who care for patients with life-limiting conditions to engage patients in discussions about EOL. These clinicians can assess patients’ understanding of life-limiting conditions, recognize common illness trajectories, support the expression of wishes and goals-of-care, and facilitate goal setting, decision-making and informed consent in order to support the best possible outcomes and quality-of-life.

An interprofessional primary care team could address unmet emotional, psychological, spiritual, and informational needs of patients at EOL more effectively than a physician or nurse practitioner alone because this approach provides well-rounded information from a variety of disciplines and improves access to timely EOL conversations due to the availability of more clinicians [12, 29]. However, research examining how clinicians collaborate to support EOL conversations reveals nurses and allied health clinicians are most often not engaging patients in these conversations [7, 12, 30]. Previous studies have aimed to improve the problem of low interprofessional collaboration in serious illness conversations by improving the communication training of clinicians from multiple disciplines [7, 28]. Although these interventions promoted more frequent and higher quality conversations, they did not lead to earlier conversations in patients’ illness trajectory [7, 28]. Clinicians also experienced challenges including role confusion, less trust from clinicians, exclusion from collaboration, and the perception that EOL conversations were futile [7]. The social, political, and professional conditions that created these forms of collaboration were not examined in this research, leaving gaps in understanding how social and practice structures shape collaboration.

As critical health scholars who are interested in the ways normative (i.e., dominant social rules) logics and social structures shape care, our previous work explored how biomedical norms constrain EOL conversations between physicians or nurse practitioners (e.g., patient’s most responsible providers) and frail older adults and/or their care partners in an urban Family Health Team [30]. Our findings suggest that attempts by patients or the most responsible provider to talk about decline, death, or the limits of medicine, were constrained by talk and behaviour that emphasised the possibility of living longer [30]. The logic of reversing or mitigating decline is reinforced by biomedical culture, clinical practice guidelines, and the societal expectation of longevity, making it less possible for EOL conversations to occur in primary care. This work demonstrated the importance of examining the way broad, yet often hidden forces shape EOL conversations.

In this manuscript we build on our previous analysis of ethnographic data to critically explore how interactions within a team impact EOL conversations. We previously found that while the conversations were fragmented, patient’s primary physician or nurse practitioner did engage frail older adults in goals-of-care discussions or decision-making, yet nurses and allied health clinicians did not [30]. To investigate why this pattern was observed, we explore the factors that influence the quality of collaboration in the primary care team as well as policies that govern interprofessional practice more broadly.

## Methods

We engaged in a critical ethnography using observations, document analysis, and interviews to gather in-depth information about how macro-structures, such as policy and normative assumptions, influence how a team of clinicians collaborate around EOL conversations [31–33]. Critical ethnography differs from ethnography in that it seeks not only to understand and describe the language and behaviours of a group at the micro level, but also interpret how group culture is shaped by sociopolitical structures [33]. To guide our investigation of characteristics shaping patterns of collaboration between clinicians from multiple disciplines, we drew on the Gears Model of Factors Affecting Interprofessional Collaboration (see Table 3) [34]. Like other investigators [35–37], we used this taxonomy of characteristics at the macro, meso, and micro levels to examine the quality of collaboration in this team. To link patterns of collaboration with sociopolitical structures, we also examined the governing policies of the clinic and normative assumptions within the interviews, observations, and policies [20, 38].

### Setting and recruitment

The Family Health Team (referred to as the clinic) we studied is part of a larger academic teaching hospital located in Ontario, Canada that is governed by a physician board. This teaching hospital has an existing interprofessional education program, including access to structured student and staff interprofessional education, and dedicated resources. Additionally, there was at least one champion within the clinic who acts as a representative for the institutional interests in effective collaboration. The clinicians also received professional development on EOL conversations.

The clinic is comprised of over 20 staff family medicine physicians and several nurse practitioners who are nurses with ‘extended class’, meaning they have received graduate university education allowing them to order tests, diagnose, and prescribe. The clinic also includes nurses and allied health clinicians such as social workers, an occupational therapist, physiotherapist, pharmacists, and dieticians. This team works with patients of all ages, including frail older adults, and patients from diverse socio-economic and racialized backgrounds.

We used purposeful sampling which allowed for recruitment of participants who could provide rich information about the topic of inquiry and allowed for in-depth understanding of an issue with the results being transferable, rather than generalizable [39]. To participate, clinicians had to care for patients who they considered to be mildly to severely frail on the Clinical Frailty Scale [40] (used for recruitment, not care). A senior physician at the clinic facilitated clinician recruitment.

### Clinician participants

We employ certain terminology to refer to the clinicians in our study. The term ‘allied health clinician’ refers to a social worker, physiotherapist, and occupational therapist. The term ‘nurse’, refers to registered and registered practical nurses who share similar scopes of practice, as opposed to ‘nurse practitioners’ whose scope of practice resembles family medicine physicians. ‘Medical professionals’ refers to both nurse practitioners and physicians who act as the most responsible provider to patients in the clinic. Grouping participants together is important to protect anonymity.

Twenty (*n* = 20) clinicians participated in this study: 10 medical professionals (8 physicians + 2 nurse practitioners); 4 nurses (including both registered nurses and registered practical nurses); 4 allied health clinicians (including 1 social worker; 1 occupational therapist; 1 pharmacist; 1 physiotherapist); and 2 medical students. One clinician who was approached declined to participate and one clinician withdrew after being observed due to increasing work demands (see Table 1 for demographics of clinician participants). All participants involved in the study provided informed written consent prior to their engagement in the research process, and assent was obtained during each research encounter.

**Table 1:** Demographics of participants

**Participant #**	**Profession**	**Race & Gender**	**Yrs. working with frail older adults**	**Yrs. working in primary care**
001	Medicine*	White man	10 + yrs	10 + yrs
002	Medicine*	White man	6-10yrs	6-10yrs
003	Allied health	Woman*	10 + yrs	1-5yrs
004	Allied health	Woman*	10 + yrs	10 + yrs
005	Nursing	BIPOC* woman	1-5yrs	1-5yrs
007	Medicine*	White woman	6-10yrs	6-10yrs
009	Nursing	BIPOC* woman	10 + yrs	10 + yrs
010	Allied health	Woman*	10 + yrs	6-10yrs
012	Medicine*	White woman	1-5yrs	1-5yrs
013	Medicine*	BIPOC* man	6-10yrs	6-10yrs
015	Allied health	Woman*	6-10yrs	10 + yrs
022	Medicine*	White man	6-10yrs	10 + yrs
023	Nursing	BIPOC* woman	6-10yrs	1-5yrs
025	Medicine*	White woman	10 + yrs	10 + yrs
026	Medicine*	White woman	10 + yrs	10 + yrs
029	Medicine*	BIPOC* woman	10 + yrs	10 + yrs
030	Nursing	White woman	10 + yrs	10 + yrs
033	Medicine*	BIPOC* woman	1-5yrs	1-5yrs
034	Medical student	White man	less than 1 yr	less than 1 yr
035	Medical student	BIPOC* man	less than 1 yr	less than 1 yr

*Nurse practitioners and physicians are grouped together as “medicine” profession to protect anonymity. *BIPOC Black, Indigenous, people of colour. *The race of allied health clinicians is not reported to protect anonymity. Total hours of observations not tallied due to multiple participants being observed at the same time

### Data production

We utilized several data production strategies: observations of clinicians in their day-to-day activities; one-to-one semi-structured interviews; and document analysis. Data were produced from February – October 2019 resulting in 17 interviews with clinicians (one clinician withdrew prior to the interview, and two left the clinic) each lasting 60 minutes on average, and over 100 hours of structured observations of clinicians’ day-to-day activities excluding direct patient care. On average, each clinician was observed for 6.7 hours (min 1 h and max 13.5 h) (see Table 2 for the data production of each participant).

**Table 2:** Participant’s data production

Participant #	Profession	Observations	Interview
001	Medicine*	13.5 h	Yes
002	Medicine*	11 h	Yes
003	Allied health	10 h	Yes
004	Allied health	9.5 h	Yes
005	Nursing	8 h	Yes
007	Medicine*	11 h	Yes
009	Nursing	11 h	Yes
010	Allied health	8 h	Yes
012	Medicine*	10 h	Yes
013	Medicine*	12 h	Yes
015	Allied health	5 h	No (withdrew)
022	Medicine*	4 h	Yes
023	Nursing	4 h	Yes
025	Medicine*	4 h	Yes
026	Medicine*	4 h	Yes
029	Medicine*	2 h	Yes
030	Nursing	2 h	Yes
033	Medicine*	1 h	No (left clinic)
034	Medical student	1 h	No (left clinic)
035	Medical student	2 h	Yes

*Nurse practitioners and physicians are grouped together as “medicine” profession to protect anonymity

An observation guide was used to focus the first author’s (CC) attention in the field on people, communication, collaboration, conflict, and talk of frailty or EOL. CC wrote reflexive notes about initial impressions, decisions about what to observe and when, how participants responded to her, and ethical dilemmas such as being shown patient records that were not part of the research protocol. Interviews were conducted by CC in person at the clinic using a semi-structured interview script. Interviews were recorded and transcribed verbatim by a transcription service. CC listened to audio recordings to check the accuracy of the transcription. Transcripts were then de-identified and audio recordings deleted. The remaining data will be retained on a password protected secure server for 5 years and then deleted. To increase rigor, the team held routine meetings to discuss data production strategies, such as note taking techniques, examine decisions in the field, and explore initial analytic ideas [41].

To produce data about the structural level policies that shape primary care practice in Ontario, Canada, we conducted searches of policies and documents, and consulted with experts, including the Association of Family Health Teams of Ontario. Some governing policies such as annual funding agreements between the Family Health Team and the Ontario Ministry of Health and Long-Term Care (MOHLTC) were not publicly available. The documents we analyzed include: 1) the Ontario Palliative Care Competency Framework; 2) Primary Care Performance Measurement Framework for Ontario 2014; 3) Physician Service Agreement 2012; 4) Health Quality Ontario – Primary Care Performance in Ontario 2019; and 5) Family Health Team Accountability Reform Application Package 2014–2015. These policies were included because of their central role in governing primary care through the enforcement of practice competencies, funding priorities, performance measures, and clinical accountabilities.

### Data analysis

Our analysis began with data simplification using the Gears Model (see Table 3) [34]. This framework was used as a coding structure to code observation notes, interview transcripts, and document data to draw our attention to factors in the data such as beliefs and formal information structures that shape collaboration [34, 42]. We then examined how the characteristics, captured by each of the code, impacted the way nurses and allied health clinicians collaborate in EOL conversations. These codes were then sorted by characteristics that support EOL conversations such as knowledge, and by barriers to EOL conversations such as prioritizing biomedical assessments. Secondly, we critically examined the coded data to understand the assumptions and logics influencing collaboration. We accomplished this by paying attention to patterns of language and actions that revealed the underlying assumptions [20]. This final progression of coding resulted in the creation of a number of themes, which we present in our findings.

**Table 3:** Gears model of factors affecting interprofessional collaboration

**Level of influence**	**Characteristics**
**Individual**	Beliefs
**Micro/practice**	Team structure (e.g., champion, size, infrastructure)
	Social processes (e.g., conflict resolution, open communication, supportive colleagues)
	Formal processes (e.g., team meetings, decision-making, group problem solving, role clarity, shared responsibility)
	Team attitudes (e.g., feeling part of the team, support for innovation)
**Meso/institutional**	Organizational culture (e.g., respect, hierarchy, focus on efficiency and achievement)
	Formal information systems (e.g., electronic medical records, quality improvement)
**Macro/structural**	Clinician education and training (e.g., competencies)
	Economics (e.g., funding, remuneration)
	Legal and regulatory context (e.g., accountability)

(Mulvale, Embrett, & Razavi, 2016) [34]

## Results

Our findings suggest that nurses and allied health clinicians, including a social worker, physiotherapist, and occupational therapist, have the knowledge, skills, and willingness to facilitate EOL goals-of-care and decision-making discussions with frail older adults. However, most of these clinicians did not engage in EOL conversations within this setting. We argue that forces at the practice, organizational, and structural levels constrain nurses and allied health clinicians’ practice. These constraints can be traced to neoliberal-biomedical ideas that normalize and prioritize biomedical effectiveness and efficiency. These ideas converge to impact team collaboration in particular ways that limit nurses and allied health clinicians involvement in EOL conversations.

### A culture governed by neoliberal-biomedicine

Primary Care Performance Measures in Ontario assess access, patient-centredness, integration, effectiveness, focus on population health, efficiency, safety, and appropriate resources (see Additional file 1). From our examination of these measures, we suggest the governance of primary care in Ontario, at the time of the study, is shaped by neoliberal-biomedical logics. Neoliberal-biomedicine refers to the way pervasive social norms, such as efficiency and cost containment, are intertwined to produce certain effects on care. We observed how efficiency relating to cost and speed as well as individualism, promotes individual responsibility for health and construes freedom as choice.

Performance indicators for access mostly emphasize quantity of, and speed at which patients are seen by a physician or nurse practitioner, while also assessing the reduction of costly acute care services. For example, one measure evaluates, ‘the percentage of patients who report that they were able to see their physician or nurse practitioner on the same or next day’. Performance indicators for integration tend to emphasize cost containment by discouraging duplication of services and the prevention of hospitalizations, with little emphasis on non-medical, social, or community care. Indicators for population health tend to promote individual responsibility for the prevention of illness by evaluating the percentage of patients who have engaged in health screenings and certain lifestyle choices. For example, one measure evaluates ‘the percentage of female grade-eight students who have completed vaccination against human papillomavirus’.

Efficiency logic intersects with biomedicine particularly through the promotion of evidence-based practice that not only justifies the use of public funds but promotes cost-saving health prevention initiatives. When we refer to biomedicine in this context, we are most interested in biomedical dominance and the control over what constitutes legitimate “healthcare” and who can practice it. Within the performance measures, we found indicators for effectiveness often promote the appropriate screening and treatment of chronic illness emphasizing that illnesses can and should be treated.

An objective of the performance measure is to assess patient centredness. A system-wide objective of patient centredness based on best practice should encourage collaborative relationships between clinicians and patients that prioritize patients’ values, goals, beliefs, and needs. However, patient-centred care is measured by access to biomedical care and individual choice, thereby reproducing logics of biomedical effectiveness and individualism, not patient-centredness. For example, measures evaluated ‘spending enough time with’ ‘involvement in decision-making’, and ‘an opportunity to ask questions’. Our overall analysis of performance measures suggests that neoliberal-biomedicine, particularly the logics of biomedical effectiveness and efficiency are evident at the structural level. We also found this ideology exerts, to various degrees, influence on beliefs and actions at the organizational and practice levels shaping team collaboration.

### Nursing and allied health knowledge and skills to support EOL conversations

Nurses and allied health clinicians had consistent and clear beliefs about when to initiate EOL conversations, what to discuss, and how to discuss it. To describe when it is appropriate to start having EOL conversations, the nurses and allied health clinicians spoke of conditions that signify the body is deteriorating, such as being frail, getting a diagnosis of a terminal illness, or a decrease in health status. A clinic nurse explains:

The right time? If the person is frail. … terminal illness. … you watch gradual decline. If they are already declining, you want to talk to them before dementia. … if there is a gradual decline. Yeah, if something is changing. (Nurse 009)

Several nurses and allied health clinicians affirmed practical things should be discussed during EOL conversations such as choosing a substitute decision-maker, talking about preferences for interventions at the EOL, and asking about the patient’s desired place of death. One allied health clinician shares the types of questions that are important to bring up with a medically frail older adult:

Have you also thought about (pause) in the event that (pause) you know, that your health starts to fail, you’re not able to make decisions for yourself, you know, what might be your concerns and what things could you put in place to help in that scenario? (Allied Health Clinician 004)

The nurses and allied health clinicians also spoke about the importance of person-centredness during EOL conversations, which extends beyond offering and exploring choice about EOL care. These participants believe it is important to elicit patients’ goals and wishes at the EOL, including what is important to them and what they are hoping to accomplish before they die. They emphasized how the personhood of the patient should be discussed, not just their physical body. A nurse explains:

We might think the focus is physically what you want done, but for some people, the focus maybe more of like a spiritual aspect … so just in sharing or asking them what was the goal of your loved one, what was their desire for death … anything they wanted to do or accomplish … just figuring out how they can live in their last days, live and die with dignity … just kind of identifying what are their wishes, what are their desires and yeah, and ensuring that we can support them in that sense. (Nurse 023)

Many of the nurses and allied health clinicians also drew on person-centredness to emphasis the role rapport plays in EOL conversations. For example, many believe EOL conversations should be initiated by someone with an existing relationship who knows the patient’s values and has their trust, which is usually their primary physician or nurse practitioner:

Usually it’s the physician that initiates it. It should be someone who the patient has a trusting relationship with too. Yeah someone that they’ve built a rapport with, I think like a healthcare professional who is understanding … who knows their situation a little bit and they’re able to have honest, open, trusting communication with them. (Nurse 023)

The nurses and allied health clinicians’ descriptions of EOL conversations highlights their overall knowledge of this complex practice. Despite this knowledge, there was agreement that nurses and allied health clinicians are not generally involved in EOL conversations, yet it could be beneficial if they played a bigger role. Some medical professionals in the team suggested the value of nurses and allied health clinicians taking on more responsibilities:

…[C]ertainly nurses and, you know, nurses ask my patients (pause) about their sex history, … they ask them about all, like all kinds of … taboo topics … So, I have no concerns about them being able to address like end of life in a, you know, a sensitive sort of open-ended…manner. … the social worker or the counsellors, yeah I think anyone could … but yeah. … I don’t think in ten years I’ve ever, like I remember anybody coming to me and being like, oh you know, like I’m the nurse, I took their blood pressure, I was talking to them about their hospitalisation and, you know, I asked a bit about who’s going to make decisions for them, that’s never happened. (Medical professional 029)

Nurses and allied health clinicians hold practice knowledge about EOL conversations that supports them to engage frail older adults in EOL conversations, yet this group rarely enacts this dimension of care. Part of the reason for this appears to be a culture characterized by certain patterns of collaboration that make it difficult for nurses and allied health clinicians to initiate and sustain EOL goals-of-care and decision-making discussions.

### Biomedical dominance and efficiency: Constraints to nurses and allied health clinicians’ practice

When examining interprofessional collaboration at the clinic, biomedical dominance is noticeable in which profession is most central to patients’ care. At the clinic, practice is structured so that nurses and allied health clinicians are positioned to provide episodic, task-based care, which limits their knowledge of, and rapport with patients, making it less possible for them to support EOL goals-of-care and decision-making discussions. Additionally, influenced by efficiency logic, the clinical team works to maintain fast-paced care, which, leads to a particular pattern of relating between disciplines, making it less possible for nurses and allied health clinicians to engage in EOL conversations. Our findings of how biomedical dominance and efficiency constrain collaboration in EOL conversations beyond physicians and nurse practitioners are organized into three sections, 1) lack of longitudinal relationships; 2) lack of collaborative decision-making; and 3) undervaluing nurses’ practice.

#### Lack of longitudinal relationships

Biomedical dominance is reproduced in the way nurses and allied health clinicians’ roles are organized in the clinic. Whereas these clinicians are relegated to short-term care, physicians and nurse practitioners foster longitudinal relationships with patients over time, which supports the facilitation of EOL goals-of-care and decision-making conservation because of their understanding of patients’ medical history and values. One of the medical professionals explains how longitudinal relationships support EOL conversations:

Having a conversation is really important, but just in kind of understanding what their [a patient’s] past behaviour is like and having conversations about their life, about their childhood, about what’s going on now, … I get a sense of who they are. And I’m not suggesting that that should replace a good [goals-of-care] conversation where you allow that person the opportunity to actually say it, but it’s a really rich (pause) in an area where you get to know people over a longitudinal thing, it’s a very rich environment to understand people’s values, wishes … what they define as quality. (Medical professionals 002)

Long-term relationships make it easier to engage in EOL goals-of-care and decision-making discussions with patients. Another medical professional agrees, and elaborates that it can be challenging for nurse practitioners and physicians to find the time to support EOL conversations, and that it would be helpful if nurses and allied health clinicians could assist in this work. However a lack of close relationships with patients makes this difficult:

[J]ust putting those first few questions out [philosophic or value-based discussions], you can’t just walk out of the room, like it turns into a longer appointment and then you’re behind … it would be nice to [have] … people to be able to talk through this kind of thing, more social workers. But if they don’t have that long-term relationship with the patient, it’s not going to go anywhere, right? So, it comes back to the same people who have the long-term relationship, who are busy, and have lots of patients (pause) like it just is not (pause) it’s not great at all. (Medical professional 026)

The way roles are designed, physicians and nurse practitioners are central to patient care as they develop continuous relationships with their patients. This is in contrast to nurses and allied health clinicians who often have longer appointments with patients, yet they generally do not know the patients well, thereby making it more difficult to engage in EOL goals-of-care and decision-making discussions. A nurse discusses how the organization of their role as task-based limits the possibility of EOL conversations:

The thing is with the clinic, it’s like more episodic and focus on one (pause) single issues. So, like just sometimes in the clinic we just do blood pressure or dressing change, or just specific task. It’s hard for me to kind of make just a decision (pause) oh the patient needs advanced care planning. … Just because I feel like I don’t see the whole picture of their health. (Nurse 005)

Because of biomedical dominance, the established pattern of collaboration is to involve nurses and allied health clinicians in task-based care which limits what these clinicians know about patients and how they contribute to their care. Without longitudinal relationships and knowledge of patient’s health history, it becomes more difficult and less likely for these clinicians to engage in EOL goals-of-care and decision-making discussions.

#### Lack of collaborative decision-making

Nurses and allied health clinicians are most often involved in patient care because of a request or referral from the patient’s physician or nurse practitioner. Nurses are most often involved in patient care with in-the-moment task-based requests, whereas allied health clinicians often receive an electronic referral requesting specific types of support. In describing the organization of their role, an allied health clinician (003) states “I’m mandated to see each patient only once. I’m in a consultant role”. Another allied health clinician further explains the care they provide and how they communicate with physicians and nurse practitioners:

I see people two to three times, and then discharge them to services that can be longer-term. I always write to the referring provider with a note about history, clinical presentation and plan and goals. I usually get a note back and we dialogue this way. (Allied Health Clinician 010)

When requested to provide care, nurses and allied health clinicians use their specialized knowledge to support patients through assessments and access to other services, as opposed to providing long-term therapy, planning, or follow-up care. The biomedical dominance that shapes the organization of allied health clinicians’ work also leads some of these clinicians to feel excluded from shared decision-making and collaboration in patient care. An allied health clinician explains:

Some physicians do not referral at all. They maybe don’t know what I do. (pause) or they might have myopic focus on medicine and pay less attention to the psycho-social issues and ways we can help. … It’s not as collaborative and cohesive as could be … There’s no mechanism for that collaboration really. (Allied Health Clinician 004)

Nurses and allied health clinicians occupy a supportive if not marginalized position in the clinic. Biomedical dominance makes the work and knowledge of physicians and nurse practitioners central to patient care with there being few opportunities to include nurses and allied health clinicians in shaping patient care to a similar extent. Additionally, by design of the clinical workflow and the allocation of clinical responsibilities, nurses and allied health clinicians provide episodic, referral-based care, which has consequences for their ability to engage in EOL goals-of-care and decision-making discussions.

#### Undervaluing nurses’ practice

Observation notes by first author CC, detail the constant work done by the team to provide efficient, fast-paced care. An example of this is the way it was noted that team members are friendly and polite but walk quickly around the clinic going from one task to another, rarely stopping. An excerpt of observation notes exemplifies this:

Observing a physician: … He comes out of his room walking towards the nursing station. A nurse is there and gives an update about a blood pressure reading. “That’s great, thank you [name]!” he says and puts a piece of paper in a mailbox. He turns back to his room, quickly sits down at the computer, pulls something up on the screen, skims it and then walks quickly to the waiting room to get his next patient.

Efficiency logic is also reproduced in the way some clinicians speak about their roles. This is particularly true for physicians who see their time as a resource that needs to be used efficiently and fairly. Consider the following two comments of medical professionals on the culture of efficiency:

It’s realizing that as much as you want to spend 90 minutes with a patient, that comes at the expense of your other patients. So, you have to balance that time in the room with this patient, against the patients that are outside in the waiting room who you also need to see. (Medical professional 013)

We think of our time as a resource. And you know, if I spend an hour with a patient, that means there’s three other patients I don’t see, so my job is to create access. (Medical professional 001)

Efficiency logic influences the collaboration between nurses and physicians in such a way that limits nurses’ involvement in EOL conversations. At the heart of this efficient primary care clinic is the 15-min appointment with a physician, with 30–45 min appointments for some complex frail patients. For physicians to see a new patient every 15–30 min, they often require help from nurses. A participant explains:

I admit, our physicians think about their practice, their population, how can you help me with X … they just think about getting through their day with these patients and this problem in front of them, because they’re too busy to think any other way. So, they’re like, ‘I want a nurse to help me with all these people.’ (Medical professional 007)

The expectation for efficiency shapes the role of nurses to help physicians with their patient care, rather than cultivate their own forms of practice with patients. Nursing roles at this clinic most often include giving immunizations, measuring vital signs, assessing infants, doing wound care, administrative tasks, and healthcare or team organization. A nurse comments on their role and collaboration with physicians:

I feel as a nursing scope of practice, … we have well-baby assessments [developmental and safety screening for infants and toddlers] or help with physicals, dressing change, those kinds of things … it just really depends on the need of the clinic. If (pause) … the family doctor … maybe they are too busy with medical care … it’s important for a nurse … then [to] help the doctors. (Nurse 005)

Physicians often request in-the-moment support from nurses, which would catch them mid-task in their clinical care. This form of collaboration prioritizes efficient biomedicine but leads to interruptions of nursing work. Interruptions indicate an undervaluing of nursing practice as a cultural norm and makes it less possible for nurses to have EOL goals-of-care and decision-making discussions because they are less likely than other clinicians to have uninterrupted time. A nurse explains:

I’ve worked really hard on if my door’s closed then there’s a reason for that, and my colleagues know that. … I would hope that…my colleagues and management would see it as a worthwhile time for me to spend time with these people, whoever needs to have that [EOL] conversation, and would respect that. … but I do have interruptions at times, and it does complicate that conversation. … interruption is a big one. So even though we went, we just had that big spew about what I try to set the tone for, I still get interrupted. (Nurse 030)

While this pattern of relating is normalized due to neoliberal-biomedical logics, some nurses at this clinic resist it. A group of the nurses worked with management to stop interruptions to their work. Management announced a written nursing request system that the team is meant to use instead of interrupting. A medical professional explains why this is important as captured in the following observation note:

A physician said he had to take a lengthy history and I [author CC] asked if he often would ask a nurse to do it. He said that’s a good question and shut the door. He said we’re in a difficult time. There has been the introduction of the medical communication form that just happened … He said it’s been years in the making. … The nurses do not want to do menial tasks. Workload is high and they want to practice to their full scope. It is not okay to ask nurses to do histories or pre-screening because they have other more important work to do. He says if he’s behind, he can ask nurses to help him out, but that should not be a regular thing.

However, despite this new communication system, interruptions continued. This was captured in author CC’s observation notes:

A medical resident comes out of his room in a hurry asking where is nursing? I’m [Author CC] the only person there and say, ‘they all seem occupied’ and gesture to the closed doors of the nursing offices. He looks around at all the doors and then a nurse comes out of her room with a blood pressure machine putting it back where it is stored. The resident tells her he needs help with vaccinations. The nurse pauses, seeming unsure of what to do. She turns back to her room saying she is with a patient right now and instructs him to write a communication note. He pops into another nurse’s office and asks for help.

Neoliberal-biomedical logics normalizes a particular way of collaborating in this clinic that prioritizes efficient fast-paced medical care. This logic leads to the organization of nurses and their work to be task-oriented and driven to meet the needs of the clinic but undervalues nurses’ independent practice and expertise. This context and culture of collaboration is one of the ways nurses are constrained in their ability to engage in EOL goals-of-care and decision-making discussions.

## Discussion

Our analysis suggests that the distribution of tasks and roles in this Family Health Teamis shaped by neoliberal-biomedical logics that normalize and prioritize biomedical effectiveness, biomedical dominance, and efficiency, thereby limiting interprofessional collaboration for EOL goals-of-care and decision-making discussions. Biomedical effectiveness and dominance prioritize the role of biomedicine in sustaining patients’ physical health, preventing decline and death, and controlling what counts as healthcare and who can practice it [21, 43, 44]. Efficiency prioritizes speed and minimizing costs [16, 22, 45]. Together these logics create a culture that prioritizes the work of physicians and nurse practitioners while normalizing limited collaboration with nurses and allied health clinicians and restricting their practice to providing episodic task-based care that supports biomedical efficiency rather than drawing on their own professional expertise. This culture and its patterns of relating limit the possibility of nurses and allied health clinicians’ engagement in EOL conversations. Our findings align with a small but growing body of work that draws attention to the way relationships of power operate on interprofessional collaboration [46]. However, we are the first to apply this type of critical analysis to ethnographic data to explicate the way relationships of power limits collaboration for EOL goals-of-care and decision-making discussions in primary care.

Our findings suggest the barriers to nurses and allied health clinicians’ involvement in EOL conversations are less related to skills and knowledge and more rooted in normative logics that shape the way primary care service delivery is structured and evaluated. While we recommend strategies to improve interprofessional collaboration for EOL conversations be targeted at the structural level, our data does highlight some possible gaps in knowledge about EOL conversations for nurses and allied health clinicians. For example, when asked about what to discuss during EOL conversations nurses and allied health clinicians rarely mentioned the importance of exploring patients’ understanding of their illness and using patients’ goals to guide decision-making. Some resources could be directed at clarifying scopes of practice in relation to EOL conversations and providing instruction on how to engage in robust EOL goals-of-care and decision-making discussions in primary care.

Neoliberal-biomedical logics are present in the way clinicians’ roles and responsibilities are governed in primary care, with the work of physicians and nurse practitioners being organized as central to all patient care, and nurses and allied health clinicians being mandated to provide episodic care with little long-term relationship development with patients. Structuring care this way supports biomedical effectiveness and efficiency. Neoliberal-biomedical logics are also present at the practice level with nurses and allied health clinicians being less involved in decision-making about patient care and ownership of care.

Hierarchies between clinicians from different disciplines is well documented, especially between nurses and physicians [47]. There is a long history of medicine expecting obedience from nursing, with nurses being expected to act as physician’s eyes and hands [47, 48]. Nurses have often been treated as “physician’s assistants” who perform manual labour at the direction of physicians, rather than having their own practice, forms of knowledge, and expertise [48, 49]. A similar disregard for the expertise of social work and occupational therapy exists with their knowledge base and clinical effectiveness often being questioned within interprofessional medical teams [50]. Despite policy shifts towards interprofessional collaboration in the provision of primary care, physicians often remain the “de facto” leaders of these teams – a trend that disrupts collaboration with little evidence to support this hierarchy [44]. To curb biomedical dominance in primary care, foster collaboration, and embrace overlapping roles, changes to funding and governance are needed [51, 52].

We argue that for primary care nurses and allied health clinicians to become involved in EOL conversations, their expertise needs to be valued, they need to be equal members of the team who share in decision-making about what care is needed, have professional autonomy, and be able to develop longitudinal trusting relationships with patients. Other research outside of primary care supports our findings. Research has found that to support EOL conversations nurses and allied health clinicians should be integral members of the team who share responsibility for making decisions about patients’ care [53, 54]. A lack of shared decision-making disrupts the ability of nurses’ and allied health clinicians’ to use their expertise, which often involves a more holistic approach that can be helpful during EOL conversations [8, 53–55].

Our analysis is also consistent with research that suggests nurses and allied health clinicians need to form trusting and ongoing clinician-patient relationships to facilitate person-centred EOL conversations [8, 53]. This was not possible at our study site due to the organization of work that inhibited participation in longitudinal care. Without sufficient knowledge of the patients, clinicians are less equipped to engage in EOL conversations because of a lack of understanding of patients’ illness history and trajectory, and needs around EOL [27, 55]. All patients do not require an interprofessional approach, but complex older patients do. We recommend primary care teams clarify nurses and allied health clinicians’ roles in EOL conversations and ensure teams are aware of, and supportive of these roles to facilitate involvement in this practice and potentially increase the quality of care for these patients [27, 54, 55]. However, we also argue that without addressing the influence of neoliberal-biomedical logics on the organization and delivery of care, nurses and allied health clinicians will likely continue to be excluded from this practice.

Most studies examining interprofessional collaboration have done so at the micro interpersonal level, ignoring the structural-level characteristics that impact collaboration [46, 51]. Our findings underscore the importance of analysis of structural characteristics as well as dominant ideologies and their influence on collaboration. While there may be strategies at the practice level to support team collaboration in primary care, such as regular team meetings, sharing responsibility for patient care, role clarity, and non-hierarchical team building [37, 56], we believe these strategies are less impactful if governing logics are left unexamined and unchanged.

Bourgeault & Mulvale [51] suggest the “caring” work of non-medical primary care clinicians such as nurses, social workers, and occupational therapists who promote well-being, fulfilling occupations, and coping, among other things, is less valued because its outcomes are more challenging to quantify than biomedical work [18, 21, 44]. We found this marginalization and devaluing of the work of nurses and allied health clinicians in our study in the way the clinic was organized to allow physicians and nurse practitioners to control who provides care and how, which resulted in nurses and allied health clinicians having limited involvement in patient care including in EOL conversations. These findings point to the importance of examining and modifying primary care quality indicators in ways that value the work of all team members and patient-centred care.

Another reason biomedical dominance remains entrenched in primary care includes funding models shaped by neoliberalism [22, 57]. Within neoliberal reforms, funding priorities are often focused on managing chronic illness, reducing cost by reducing hospital admissions, and supporting physician-owned primary care practices [17, 18, 22, 57]. Primary care clinics’ funding is often controlled by incorporated businesses governed by a physician-board that makes organizational and service delivery decisions [58]. Research from Ontario, where our study took place, has linked funding agreements such as those of Family Health Teams, to decreased interprofessional collaboration and minimal delegation of tasks to nurses and allied health clinicians [52]. We recommend primary care teams interested in cultivating team collaboration examine the influence of funding models, and work to make meaningful changes that support more collaboration for complex patient care including EOL conversations.

In Canada and internationally, research has examined how neoliberal-biomedical logics govern healthcare policy, institutional governance, and direct care in a variety of areas such as EOL care, maternal care, women’s health, addiction care, public health, emergency services, and primary care [17, 22, 59–61]. What our study adds to this scholarship is the way neoliberal-biomedical logics limit collaboration in interprofessional primary care teams, specifically in the area of EOL conversations. This is a novel and important finding because it helps to explain why taking an individual-level approach to addressing the challenge of delayed or avoided EOL conversations, specifically by educating nurses and allied health clinicians about how to facilitate EOL conversations [13, 28, 62], is unlikely to result in practice change. This is because nurses and other allied health clinicians are embedded in a biomedical culture that prioritizes biomedical effectiveness, biomedical dominance, and efficiency; until and unless these are addressed, individual-level solutions will fall short of achieving real change.

### Limitations

To protect anonymity, data from nurse practitioners and physicians were grouped together. While these two types of clinicians have similar roles at the study site, there are differences in the organization of their work that is not captured in our findings. Future research could focus on nurse practitioner led clinics to further explore this groups’ experience facilitating EOL conversations. Additionally, we were unable to determine the participants’ level of training in EOL since clinicians attended schooling at various times in various geographical locations making it impractical to review the curriculum each participant received in EOL care and interprofessional collaboration. Finally, our study site was an urban, academic, medicare funded primary care team located within a health science centre. While not generalizable, our detailed description of the setting, participants, and interactions support transferability to other contexts.

## Conclusion

Our findings suggest primary care nurses, a social worker, occupational therapist, and physiotherapist have the knowledge, skills, and inclination to engage frail older adults in EOL conversations. However, they are constrained in their ability to do this by specific patterns of relating that are shaped by neoliberal-biomedical logics operating at the structural, organizational, and practice level. Our study highlights the way these governing logics restricts interprofessional collaboration in primary care by shaping the distribution of tasks and roles in such a way that limits nurses and allied health clinicians’ engagement in EOL conversations. It is our hope that this study inspires future practice change research to improve interprofessional collaboration and EOL conversations by reimagining funding models and performance indicators in primary care that fully support meaningful interprofessional person-centred care for complex frail patients.

### Supplementary Information

**Additional file 1.** Primary Care Performance Measures in Ontario.

## Data Availability

The datasets generated during the current study are not publicly available due issues of privacy but are available from the corresponding author on reasonable request.

## Abbreviation

EOL: End-of-life
