# Supplementary material for: “I don’t see the whole picture of their health”: a critical ethnography of constraints to interprofessional collaboration in end-of-life conversations in primary care
Source: BMC Prim Care. 2023 Oct 28;24:225. doi: 10.1186/s12875-023-02171-w (PMC10612350; doi:10.1186/s12875-023-02171-w)
Supplement: Supplementary file 1 — Additional file 1. Primary Care Performance Measures in Ontario. [file 12875_2023_2171_MOESM1_ESM.docx]

Additional file 1: Primary Care Performance Measures in Ontario

| **Objective** | **Performance Measures** |
| --- | --- |
| **Access** | - % of total primary care visits that are made to the physician with whom the patient is rostered or virtually rostered - Proportion of total primary care visits that are made to the physician with whom the patient is rostered or virtually rostered - Percentage of people who report going to the emergency department for reasons that were potentially avoidable - Percentage of people who report going to a walk-in clinic for reasons that were potentially avoidable - % of patients who report that they have a family physician or nurse practitioner - Percentage of patients who report that they experienced difficulties obtaining required routine or ongoing primary care services from their provider over the past 12 months, for themselves, their children, elderly family members or disabled family members - % of patients who report that they were able to see their physician or nurse practitioner on the same or next day - % of patients who report that getting medical care in the evening or on a weekend or public holiday was difficult - % of patients who report that, when they call their physician or nurse practitioner with a medical question or concern during regular office hours, they get an answer on the same day - % of patients who report that they have emailed their family physician or nurse practitioner with a medical question in the last 12 months - % of patients who report that it would have been better for their health if their regular family physician or nurse practitioner had come to see them at home rather than going to their office |
| **Patient-Centredness** | - % of patients who report their family physician, nurse practitioner, or someone else in their office spends enough time with them - % of patients who report their family physician, nurse practitioner, or someone else in their office involves them as much as they want in decisions about their care or treatment - % of patients who report that their family physician, nurse practitioner or someone else in their office explains things in a way that is easy to understand - % of patients who report that their family physician, nurse practitioner or someone else in their office gives them an opportunity to ask questions about recommended treatment |
| **Integration** | - % of patients who were readmitted to a hospital following their initial hospitalization - Rate of hospital admissions for specific chronic conditions per 1,000 population, by condition (asthma, chronic obstructive pulmonary disease, congestive heart failure, diabetes) and combined - % of patients who see their primary care provider within seven days after discharge from hospital, for selected conditions - % of patients who report that the hospital made arrangements for their follow-up care with a physician or other health care professional - % of patients who report that, in the last 12 months, when receiving care for a medical problem, there was a time when test results were not available at the time of a scheduled appointment with their family physician - % of patients who report that their family physician/nurse practitioner was informed and up-to-date about the care they received from specialists - % of patients who report that their family physician/nurse practitioner was informed and up-to-date about the care they received in the hospital - % of primary care physicians who report that they receive the following information after their patients’ visits to specialists:   - A report back from the specialist with all relevant health information   - Information about changes the specialist has made to the patient’s medication or care plan   - Information that is timely and available when needed - % of primary care physicians who report that they receive notification that their patient:   - Has been seen in the emergency department   - Is being discharged from the hospital - % of primary care physicians who report that on average they receive the needed information after their patients’ discharge from hospital within:   - Less than 24 hours   - 24 – 48 hours   - … - % of patients who report delays in being notified about abnormal test results in the past two years - % of patients who report that their family physician or someone in their office helped them book appointments or coordinate the care they received from specialists over the past 12 months - % of primary care physicians who report that they or someone else in the practice provides care in the following ways:   - Managing and coordinating care for their patients after hospital discharge   - Coordinating care with social services or other community providers - Self-reported wait times for patients who were advised to see a specialist |
| **Effectiveness** | - % of patients aged 65+ on the recommended drugs (beta-blocker, angiotensin-converting enzyme inhibitor or angiotensin receptor blocker and statin) after hospitalization for acute myocardial infarction - % of patients aged 65+ with diabetes who were prescribed a statin within the past 12 months - % of patients aged 65+ with diabetes who were prescribed an angiotensin-converting enzyme inhibitor or angiotensin receptor blocker within the past 12 months - % of patients with diabetes with at least one low-density lipoprotein (LDL) cholesterol test within the past 12 months - % of patients with diabetes with two or more glycated hemoglobin (HbA1c) tests within the past 12 months - % of patients with diabetes with at least one retinal examination within the past 24 months - % of patients with diabetes for whom a physician billed the diabetes management assessment code (K030) at least once during the past 12 months - % of primary care physician who report using a flow sheet or checklist for chronic diseases - % of patients with new congestive heart failure who have a left ventricular function test - % of patients aged 65+ on the recommended drugs (beta-blocker and angiotensin -converting enzyme inhibitor or angiotensin receptor blocker) after hospitalization for congestive heart failure - % of patients with diabetes for more than a year who had a serious diabetes complication (death, heart attack, stroke, amputation or kidney failure) in the past 12 months - % of people with hypertension, heart disease or diabetes who report that they had their blood pressure checked in the past 12 months - % of patients aged 65+ newly diagnosed with hypertension who are prescribed a thiazide as an anti-hypertensive - % of patients who have a mental-health follow-up visit to a physician (primary care physician or psychiatrist), within seven and 30 days of discharge following hospitalization for a psychiatric condition - % of people who report being able to get help from a professional when dealing with emotional distress, such as anxiety or depression, in the past two years |
| **Focus on Population Health** | - % of women aged 50 to 74 who had a mammogram within the past two years - % of patients aged 50 to 74 who had a fecal occult blood test (FOBT) within the past 2yrs, sigmoidoscopy or barium enema within 5yrs or a colonoscopy within the past 10yrs - % of patients aged 50 to 74 who completed a fecal occult blood test (FOBT) in the past two years - % of women aged 21 to 69 who had a Papanicolaou (Pap) smear within the past three years - Population demographic information:   - Age   - Gender   - Income   - Education   - Location of residence   - … - % of people aged 12 and over who report smoking daily or occasionally - % of people who are obese, overweight, underweight and normal weight, based on self-reported weight and height data - % of people aged 12 and over who report being physically inactive - Prevalence rate for the four most common cancers: prostate, female breast, colon and rectum, lung - Annual rates of new cases of the following cancers   - Males: prostate, colon, lung …   - Females: Breast, colon, lung … - % of primary care physicians who report that they maintain or have access to a registry of patients with the following chronic conditions:   - Asthma   - COPD   - CHF   - … - % of people who report being told that they have the following conditions:   - Asthma   - Chronic lung disease   - Cancer   - Depression, anxiety, etc.   - … - % of people who report having a discussion within the past two years with their health care provider regarding the following health behaviours/risk factors:   - A healthy diet and healthy eating   - Exercise or physical activity   - … - % of healthy neonates who had a follow-up appointment with a primary care provider within one week after birth - % of recent mothers who report breastfeeding or trying to breastfeed - % of women who had live term births who exclusively breastfed at the time of discharge from hospital - % of women who gave birth and had a prenatal-care visit in the first trimester - % of primary care physicians who report that they offer the following services in their practice:   - Prenatal care   - Intrapartum care   - Postpartum care - % of 13-year-olds who received one dose of the quadrivalent meningococcal conjugate vaccine on or before their 13^th^ birthday - % of school children aged 7 years who are fully vaccinated against diphtheria, tetanus and polio and measles mumps and rubella - % of female grade-eight students who have completed vaccination against human papillomavirus - % of grade-seven students who have completed vaccination against hepatitis B by the end of grade seven - % of people who report having a seasonal flu shot |
| **Efficiency** | - Per-capita health care expenditures by category:   - In-patient hospitalization   - Same-day surgery   - ED visits   - Visits to dialysis clinics   - Visits to cancer clinics   - Ontario Drug Benefit (ODB)   - Rehabilitation   - Complex and continuing care   - Home care services   - OHIP physician billings, including most of the shadow-billings   - OHIP lab claims   - OHIP non-physician billings   - FHO/FHN capitation   - Long-term care   - Admissions to designated mental-health beds   - Assisted Devices Program (ADP) - % of primary care physicians who report being able to generate the following patient information with their current medical records system list of patient by:   - Diagnosis   - Laboratory results   - Due or overdue for tests or preventative care   - All medications or taking a particular medication   - Clinical summaries - % of primary care physicians who report being able to electronically exchange the following with other physicians outside their practice:   - Patient clinical summaries   - Laboratory and diagnostic tests - % of primary care physicians who report using electronic records instead of paper charts to enter and retrieve patient clinical notes - % of primary care physicians who report using the following technologies in their practice:   - Electronic ordering of laboratory tests   - Electronic alerts or prompts about a potential problem with drug dose or interaction   - Electronic referring to specialists   - Electronic prescribing of medication - Per-capita health care expenditures by category:   - Inpatient hospitalization   - Same-day surgery   - ED visit   - … - % of patients who report a time in the past 2yrs when physicians ordered a medical test they felt was unnecessary, because it had already been done - % of primary care physician who report that they routinely give patients with chronic conditions written instructions on how to manage their own care at home |
| **Safety** | - % of patients who report that, in the past 12 months, they had a review and discussion with their primary care provider of prescription medications they are using - % of patients who are using two or more prescription medications who report a clinician gave them a written list of all their prescription medications - % of patients who believe a medical mistake was made in their care during the past 2yrs - % of patients who report that, in the past 12 months, a health care provider explained the potential side effects of any medication that was prescribed - % of patients who report that the health professional involved told them a medical error had been made in their treatment - % of patients who report having a negative reaction to a medication prescribed by their primary care provider that resulted in a visit to the hospital in the past two years |
| **Appropriate Resources** | - % of primary care physicians who report being able to electronically transfer prescriptions to a pharmacy - Average weekly hours that primary care physicians report spending on the following activities:   - Direct care with and without teaching   - Teaching without direct care   - Indirect patient care   - Health facility committees   - Administration   - Research   - Managing their practice   - Continuing education   - Other - Average frequency of use and impact of continuing professional education activities on primary care physicians’ practice - % of primary care physicians who report that the following factors are increasing the demand for their time at work:   - Aging patients   - Complexity   - Chronic disease   - Patient expectations   - Administrative workload   - Lack of availability of services   - Medical liability concerns   - Other - Average number of full-time-equivalent physicians working in primary care practices - Average number of full-time-equivalent non-physician providers working in primary care practices - Average # of patients that physicians report taking care of in their practice - % of primary care physicians who, during the last year:   - Used any locum tenens   - Personally provided locum tenens services for another physician - % of time physicians report spending in face-to-face contact with patients in a typical week - Primary care physicians’ average hours of medical practice per week - Percentage of income that primary care physicians report spending on overhead - Percentage of primary care physicians who report that they receive information on how the clinical performance of their practice compares to other practices:   - Routinely   - Occasionally - % of primary care physicians who report that they review some areas of clinical performance against targets, at least annually - % of primary care physicians who report that their practice routinely receives and reviews data on the following aspects of their patients’ care:   - Clinical outcomes   - Surveys of patient satisfaction and experiences   - Patients’ hospital admissions or emergency department use   - The frequency of ordering diagnostic tests   - The frequency of various conditions   - The frequency of referrals to specialists/specialized services - % of primary care physicians who provide a broad scope of primary care physician services |
| Information sources: A Primary Care Performance Measurement Framework for Ontario 2014; Physician Service Agreement 2012; Health Quality Ontario - Primary Care Performance in Ontario 2019; and Family Health Team Accountability Reform Application Package 2014-2015. | |
